# First Report of Evolut Pro+ Transcatheter Aortic Valve-in-Valve in a Degenerated Lotus Valve

**DOI:** 10.1016/j.shj.2023.100229

**Published:** 2023-11-11

**Authors:** Thorald Stolte, David Winkel, Philip Haaf, Thomas Nestelberger

**Affiliations:** a Department of Cardiology and Cardiovascular Research Institute Basel (CRIB), University Hospital Basel, University of Basel, Basel, Switzerland; b Department of Health Sciences and Technology, Swiss Federal Institute of Technology, Zurich, Switzerland; c Department of Radiology, University Hospital Basel, University of Basel, Basel, Switzerland

**Keywords:** Functional computed tomography, Transcatheter aortic valve implantation, Valve-in-valve, Valve prosthesis degeneration

## Abstract

The increasing use of transcatheter aortic valves in patients with aortic stenosis has led to a higher number of valve-in-valve procedures due to gradual valve degeneration. We present a case of a 72-year-old woman who received transcatheter aortic valve implantation (TAVI) using a Lotus valve due to severe aortic stenosis, which showed valve degeneration several years after the initial procedure. After heart-team discussion, TAVI-in-TAVI was planned using an Evolut pro+ valve, which allowed for full coverage of the Lotus valve and maintenance of coronary flow, resulting in a well-functioning valve with no regurgitation and normal performance. Despite high-risk anatomic features, valve-in-valve using an Evolut pro+ in a degenerated Lotus valve is feasible and overcomes pitfalls such as entanglement or coronary obstruction.

## Introduction

The use of transcatheter aortic valves in patients with severe aortic stenosis and low to intermediate surgical risk has substantially increased over the last few years. Gradual valve degeneration results in the need for increasing valve-in-valve (VIV) procedures.^1^

## Case Description

In 2017, a 72-year-old woman received transcatheter aortic valve implantation (TAVI) using a 25-mm Lotus valve (Boston Scientific, Marlborough, Massachusetts, USA) due to severe aortic stenosis. Postprocedural transthoracic echocardiography (TTE) showed a well-functioning valve (mean gradient [MG] 13 mmHg, Doppler velocity index 0.31, left ventricular ejection fraction [LVEF] 30%, no regurgitation). Several months later, LVEF improved to 55%.

**Figure 1:**
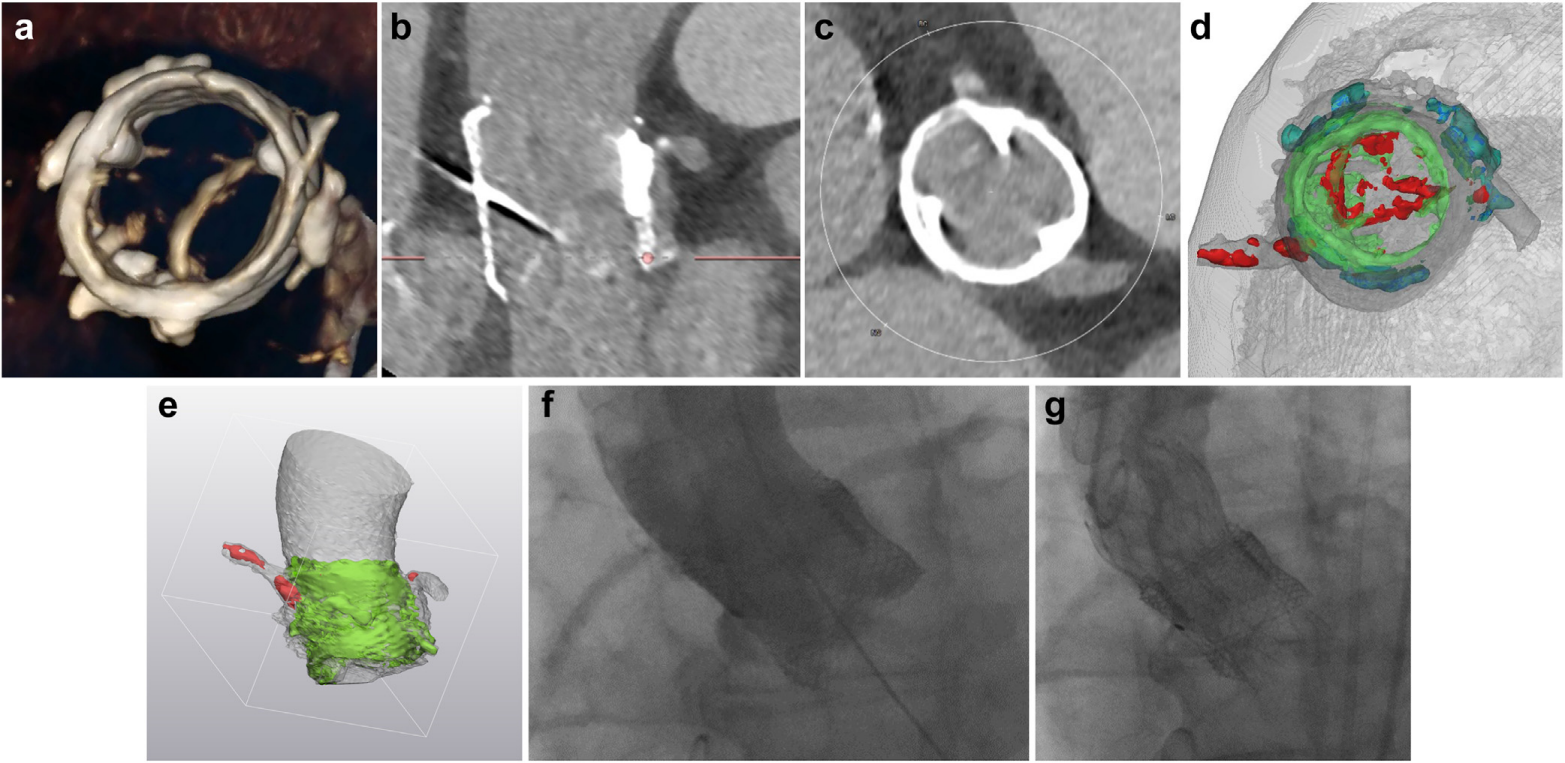
(a) 3D Cardiac computed tomography angiography (CCTA) of the Lotus prosthesis with thickening and immobility of one leaflet as well as reduced separation of all three leaflets without signs of a valve-thrombus or pannus-formation. (b and c) CCTA illustrating the relationship between coronary ostia and the Lotus valve. (d and e) 3D visualization of the aortic root and ascending aorta. (d) View through the aortic lumen from above with the 25 mm Lotus valve (in green), the calcified leaflets and coronary calcifications (in red), calcified, native aortic leaflets (in blue), and surrounding myocardium/vascular structures (in grey). (e) Modified anterior view, demonstrating that both the right coronary artery (RCA) and left main (LM) origin were partially covered by the 25 mm Lotus valve. (f and g) Peri-interventional fluoroscopy showing normal blood flow and perfusion of coronary vessels without signs of relevant regurgitation preimplantation.

In 2022, she presented with shortness of breath (NYHA III) and signs of biventricular cardiac decompensation. TTE revealed a severe low-flow, low-gradient aortic stenosis (MG 35 mmHg, LVEF 30%, aortic valve area 0.8 cm^2^, indexed stroke volume 0.32 ml/m^2^). Cardiac computed tomography angiography and a 3D visualization showed severe degeneration of the valve and high-risk features for coronary obstruction (Figure 1a-e, Supplemental Video 1). After heart-team discussion, TAVI-in-TAVI was planned using an Evolut pro+ 26 mm valve (Medtronic, Minneapolis, Massachusetts, USA) after predilation using a 18 mm True Dilation balloon (Bard Vascular Inc, Tempe, Arizona, USA; Figure 1e-g, Supplemental Video 2) based on the minimal diameter inside the Lotus valve. Angiographic assessment showed normal coronary flow and no relevant regurgitation; postprocedural TTE also revealed no regurgitation and normal performance of the valve (MG 9 mmHg, Doppler velocity index 0.36, LVEF 33%, Supplemental Videos 3 and 4). The patient was asymptomatic 1 ​month after the procedure. Oral anticoagulation with Apixaban was restarted after the procedure.

## Discussion

While surgical explantation and reimplantation of TAVI valves are associated with high perioperative risks and worse outcomes, VIV is associated with risks such as coronary obstruction or under-expansion.^2^ In our case, the high stent frame of the Lotus valve above the sino-tubular junction and the very low distance between the valve and the coronary ostia was associated with high risk for coronary obstruction. Chimney stenting was not an option given the tight stent struts of the valve. Leaflet modification was considered, the severely calcified leaflets of the Lotus valve with a higher risk of embolization, and given that the valve was partially commissural aligned, it was decided not to perform it. As the patient already had a pacemaker, we aimed to deploy the valve deeper to ensure the skirt of the Evolut pro+ valve was not affecting coronary flow. Prior case reports with other valve types in degenerated Lotus valves resulted in high gradients^3^ (Sapien S3, Edwards Lifesciences, Irvine, California, USA) or entanglement of the upper crown of an Accurate neo valve^4^ (Boston Scientific) within the stent struts of the Lotus valve. The Evolut pro+ valve allows for full coverage of the leaflets of the Lotus valve, maintenance of coronary flow given the ability to achieve commissural alignment and the large stent cells, as well as repeated repositioning.

## Conclusion

Despite high-risk anatomic features, VIV using an Evolut pro+ in a degenerated Lotus valve is feasible while overcoming pitfalls such as entanglement or coronary obstruction.

## Consent Statement

Patient consent was obtained for publication of this report and accompanying images.

## Funding

The authors have no funding to report.

## Disclosure Statement

T. Nestelberger has received research support from the Swiss National Science Foundation (P400 pm_191037/1), the Prof Dr Max Cloëtta Foundation, the Margarete und Walter Lichtenstein-Stiftung (3MS1038), and the University Hospital Basel as well as speaker honoraria/consulting honoraria from Edwards Lifesciences, Siemens, Beckman Coulter, Bayer, Ortho Clinical Diagnostics and Orion Pharma, outside the submitted work. All other authors have no conflict of interests.

## Supplementary Material

Supplemental Video 1 CCTA showing the severe degeneration, calcification and reduced leaflet separation of the valve.

Supplemental Video 2 Peri-interventional fluoroscopy pre-implantation.

Supplemental Video 3 Peri-interventional fluoroscopy post-implantation showing absence of relevant regurgitation, as well as maintained blood flow and perfusion of coronary vessels.

Supplemental Video 4 Post-interventional transthoracic echocardiography showing no relevant stenosis or regurgitation.
